# Determinants of Low Birth Weight Among Newborns Delivered at Public Health Facilities of Bishoftu Town, East Shewa Zone, Ethiopia: Unmatched Case-Control Study

**DOI:** 10.1155/2024/4873667

**Published:** 2024-07-11

**Authors:** Daniel Bekele, Balay Dhaba, Abenet Menene, Leta Hinkosa

**Affiliations:** ^1^ College of Health Science Arsi University, Asella, Ethiopia; ^2^ Bishostu Referral Hospital, East Shewa, Oromia Region, Ethiopia; ^3^ College of Health Science Wollega University, Nekemte, Ethiopia

**Keywords:** case-control, determinants, low birth weight, newborns

## Abstract

**Background:** Low birth weight (LBW) is a major global public health issue that can have a number of serious, potentially fatal health consequences. It is the most common cause of neonatal and child death in low- and middle-income countries, which also has a number of negative health effects. However, the determinants of LBWs were not yet completely recognized in Ethiopia. Thus, it is aimed at identifying the determinants of LBW among newborns delivered at public health facilities in Bishoftu town, Ethiopia.

**Methods:** A facility-based unmatched case-control study was conducted at Bishoftu town. Data were collected from mothers having newborns with birth weight < 2500 gm (cases) and 2500 to 4000 gm (controls) using a pretested questionnaire and medical record review. Lastly, Epi Info 7 to enter the obtained data, and SPSS version 21 was used for analysis. Factors in the bivariate analysis with a *p* value less than 0.25 were added to the multivariable logistic regression, where a *p* value less than 0.05 was deemed statistically significant.

**Results:**A total of 285 neonates (95 cases and 190 controls) were included in the study. Being not preeclampsia (AOR = 0.34; 95% CI: 0.13–0.88), lack of iron supplementation (AOR = 12.16; 95% CI: 5.40–27.42), preterm delivery (AOR = 7.49, 95% CI: 3.23–17.36), lack of information (AOR = 4.65, 95% CI: 1.37–15.750), and not experienced premature rupture of membranes (PROM) (AOR = 0.27; 95% CI: 0.08–0.91) were identified as statistically significant variables in LBW.

**Conclusion:** LBW was significantly influenced by preeclampsia, PROM, missing iron-folate supplementation during pregnancy, and ignorance of the warning symptoms of premature delivery during pregnancy. Therefore, reducing LBW requires a lot of work, including developing effective interventions and monitoring policies.

## 1. Introduction

The World Health Organization (WHO) defines low birth weights (LBWs) as babies who weigh less than 2500 gm upon deliver [1]. Being underweight at birth is the main risk factor for adverse health effects, such as common newborn and childhood illnesses, and is a serious global public health concern. LBW accounts for 60% to 80% of all newborn fatalities worldwide [2].

LBW is a significant contributor to prenatal mortality as well as short- and long-term morbidity in infants and children in both industrialized and developing nations. Deaths of LBW infants are 30 times more frequent than deaths of newborns of normal birth weight, and they are many times more likely to end up with long-term handicapping conditions [3]. Furthermore, they are at higher risk of perinatal death and adulthood stunting which in turn leads to the intergenerational effect of malnutrition in the affected community [4].

Globally, over 91% of LBW babies are born in low- and middle-income countries; 9.3 million of these babies are born in South Asia, and 3.1 million are born in sub-Saharan Africa. The world bears a disproportionate burden of LBW. About 25% of all LBW babies were born in Africa, with Eastern and Western Africa accounting for 11% and 14% of the total, respectively [1, 2]. Ethiopia aimed to reduce the neonatal mortality rate (NMR) from 29 per 1000 live births in 2016 to 11 per 1000 live births in 2019/2020, including LBW neonates [5]. Despite the government's aim of reducing NMR, paradoxically, it had been increased to 33/1000 live births in 2019; from this, the mortality of LBW neonates is a major public health problem. In Ethiopia, the mortality of LBW neonates ranges from 11.0% to 37.8% [6, 7].

Overall, early neonatal mortality in the Oromia region is 37 per 1000 live births, while the infant mortality rate is 60 per 1000 live births [8]. Also, according to the Ethiopian Demographic Health Survey (EDHS) 2016 estimates, 13% of neonates weighed less than 2500 gm at birth. Moreover, 14% LBW in 2005, 11% in 2011, according to mother's report, 16% of births are very small, and 10% are smaller than average [9]. In Ethiopia, the prevalence of under-five mortality ranges from 53 to 169 per 1000 live births out of this neonatal mortality which is mainly attributed to LBW accounts for the largest portion [5, 7]. Various studies conducted in different parts of developing and developed countries have found that the following factors may be risk factors for LBW: sociodemographic, medical and obstetric, nutritional, behavioral, infant, and environmental [3, 4, 10].

In East Africa, preterm babies and babies with LBW were found to account for 52% of newborn deaths. The risk of death in the first 28 days of life was also seven times higher for babies born of LBW compared to those with normal birth weight. Therefore, LBW is strongly negatively associated with infant survival [11]. More thorough research is required to identify the factors influencing LBW, as the condition continues to pose a serious public health concern in the 21st century despite the abundance of preventative alternatives and initiatives. However, there are not enough studies on the relationship between LBW risk in Ethiopia and a mother's nutritional status, food insecurity, dietary patterns, and environmental factors. Thus, the purpose of this study was to determine the contextual factors that influence LBW in the research location.

## 2. Materials and Methods

### 2.1. Study Setting and Period

The study was conducted in Bishoftu town which is found East Shewa zone Oromia region, Ethiopia, from July 1 to August 30, 2022. The town covers an area of 183 km2 and has 14 administrative kebeles. It has a total population of 234,970, of which 50% are male and 50% are female. Reproductive age group 15–49 accounts for 51,998 (pregnant women 8153) of the total population. Bishoftu town is a rapidly growing urban city both in terms of population and economy. There were six health centers, one general hospital, one private health center, and two private primary hospitals that provide reproductive health service in the town.

### 2.2. Study Design and Population

An institution-based unmatched case-control study design was employed to identify determinants of LBW among newborns delivered at public health facilities of Bishoftu town. In cases, neonates delivered with a LBW (< 2500 gm) were considered while the neonates delivered with a normal birth weight 2500 gm to 4000 gm) were considered as controls of the study.

### 2.3. Inclusion and Exclusion Criteria

#### 2.3.1. Inclusion Criteria

The inclusion criteria involve a live-born singleton baby with a birth weight of less than 2500 gm for cases and of ≥ 2500 gm for controls at birth during the study period.

#### 2.3.2. Exclusion Criteria

Newborn delivered with any congenital anomalies, greater than 4000 gm, multiple births (twin), mothers who are seriously ill during the data collection period, and those who are unable to communicate were excluded from the study.

### 2.4. Sample Size Determination

The sample was computed based on two population proportions formula by using Epi Info version 7 statistical software. It was calculated with the assumption of 95% CI, power of 80%, case-to-control ratio of 1:2, odd ratio of 2.84, and percent of controls exposed to anemia 9.1% and percent of cases exposure 22.7% [5]. Accordingly, after adding 5% for the nonresponse rate, the final sample size became 285, of which 95 cases and 190 controls were involved in the study.

### 2.5. Sampling and Data Collection Procedures

There are seven public health facilities in the Bishoftu town which provide delivery services. One hospital and two health centers were selected randomly based on 30% of the minimum criteria of representativeness. The proportional allocation of newborns to each hospital and health center was determined based on estimations obtained from the last 2 months preceding the data collection period. The cases and controls were defined according to the birth weight in the labor rooms of the facilities. Consecutive live births of less than 2500 gm in each hospital and health center were selected as cases, and two normal birth weight babies succeeding each case were selected as controls during the study period. Interviewers administered semistructured and pretested questionnaires adapted from different related kinds of literature that were used to collect the data by face-to-face interview [3, 5, 10, 11]. The questionnaire was designed to capture the sociodemographic characteristics, maternal/obstetric characteristics, maternal nutrition characteristics, toxic exposure characteristics, and medical illness characteristics. Data were collected by 3 BSc midwives and supervised by one MSc midwife who has good experience.

### 2.6. Operational Definition

Birth weight is the first weight of the newborns measured within the first hour after birth. LBW was for those newborns that weighed less than 2500 gm, while those newborns with a birth weight of 2500 gm to 4000 gm were considered of normal birth weight.

### 2.7. Data Quality Management

The data collection tool was a standardized tool taken from the national neonatal and relevant literature. The questionnaires were pretested on 5% (5 cases and 10 controls) of the calculated sample size in public health facilities ahead of the actual data collection period in order to test study measures, estimation of interview time, and clarity of tools at Chelelaka health center. One-day training was given to data collectors and supervisors. The supervisor carried out close supervision throughout the data collection time. Finally, all the collected data were checked by an investigator for completeness and consistency every day. Moreover, the data were checked during the time of management and analysis.

### 2.8. Data Processing and Analysis

After the data collection, the data were checked for its completeness every day, edited, coded, entered into the Epi Info version 7, and finally, exported to SPSS version 21 and checked for missing values before analysis. Descriptive statistics such as frequencies, percentages, summary measures, tables, and graphs were used to describe the results of the respondents. Bivariate logistic regression analysis was fitted for each exposure variable with the dependent variable to identify candidates for multivariate logistic regression. Variables with a *p* value < 0.25 were entered into the multivariate logistic regression. Adjusted odds ratio (AOR) with 95% CI and *p* value were used to measure the strength of association with LBW. Variables with a *p* value < 0.05 were declared significant. Additionally, the presence of multicollinearity was checked by employing the parameter of variance inflation factor (VIF) > 10. Finally, model fitness was checked by using the Hosmer–Lemeshow test with *p* value = 0.14.

### 2.9. Ethical Considerations

Ethical clearance letter was obtained from the Institutional Ethical Review Board (IERB) of Adama General Hospital and Medical College. Permission letters were taken from Bishoftu town Health Bureau and from the three respective facilities. Verbal consent was obtained from each mother prior to the interview.

## 3. Results

### 3.1. Sociodemographic Characteristics

A total of 285 mothers (95 cases and 190 controls) participated in the study which made the response rate of 100% for both cases and controls. The mean ± SD of maternal age among the cases was 28.4 ± 8.6 years with a range of 25–29 years. For the controls, it was 27.72 ± 6.403 years with 25–29 years of range. A higher proportion of newborns were females both in cases and controls that account for 54 (56.8%) and 92 (48.4%), respectively. For both cases and controls, mostly 94.7% of mothers of LBW babies with normal birth weight had formal education. Moreover, 76.8% of mothers among cases and 80% among controls were living in an urban setting (Table 1).

### 3.2. Obstetric and Medical Characteristics of Mothers

With regard to their pregnancy intention, 74 (77.9%) mothers of cases and 171 (90%) mothers of controls had planned pregnancy. Preterm delivery was observed among 49(51.6%) of mothers in cases and 10 (5.3%) of controls, whereas 70 (73.7%) among cases and 178 (93.7%) among controls had received information of danger signs during pregnancy. The majority of respondents, 91 (95.8%) among cases and 187 (98.4%) among controls, had ANC follow-up, while 62 (65.3%) among cases and 75 (39.5%) among controls were visited in the first trimester. Of the total participants, 31 (32.6%) cases and 20 (10.5%) of controls had experienced preeclampsia. Few mothers, 1.1% of cases had chronic hypertensive disease while 0.5% of controls had it. Moreover, mothers, 4.2% of cases and 1.1% of controls, were anemic. The majority of mothers both in cases 95% and controls 98.9% had no history of diabetes mellitus (DM) (Table 2).

### 3.3. Nutrition and Substance-Related Factors

Among study participants, the majority of 93 (97.9%) cases and 187 (98.4%) controls had no habit of smoking cigarette products. On the other hand, 46 (48.4%) cases and 88 (46.3%) controls were not drinking alcohol during the current pregnancy, but most of them occasionally drank 41 (43.2%) cases and 88 (46.3%) controls. Regarding iron supplementation, mothers who had received iron-folate supplementation less than 90 tabs were 62 (65.3%) and 38 (20.0%) among cases and controls, respectively. Mothers who had MUAC measurements of less than 23 cm were 29 (30.5%) among the cases, while the controls were 27 (14.2%) (Table 3).

### 3.4. Determinants of LBW in Bivariate and Multivariate Logistic Regression

In bivariate logistic regression, the candidate variables of mothers' age less than 20 years, gestational age less than 37 weeks, having no information about pregnancy-related danger signs, experienced any danger sign of pregnancy, experienced premature rupture of membranes (PROM), experienced preeclampsia and dose of iron supplementation less than 90 tabs, unplanned pregnancy, MUAC less than 23 cm, and had ANC visit were identified as statically significant with LBW at *p* value < 0.25 in 95% CI.

Among these variables included in multivariable logistic regression, mothers who received iron and folate supplementation less than 90 tabs during pregnancy (AOR = 12.16, 95% CI: 5.40–27.42), gestational age at delivery less than 37 weeks (AOR = 7.49, 95% CI: 3.23–17.36), had not informed about danger sign of pregnancy (AOR = 4.65, 95% CI: 1.37–15.75), had not experienced danger signs during pregnancy (AOR = 0.21, 95% CI: 0.07–0.57), had no PROM (AOR = 0.27, 95% CI: 0.08–0.91), had not experienced preeclampsia (AOR = 0.34, 95% CI: 0.13–0.88) were found to be independent determinants of LBW (Table 4).

## 4. Discussion

LBW is a serious global public health issue. Because it is the primary predictor of infant morbidity and mortality, it is given top priority. The present study revealed that the risk of LBW was higher among mothers who did not intake iron-folate supplements greater than 90 tabs during pregnancy than mothers who did take iron-folate supplementation during pregnancy. This finding is in line with a randomized controlled experiment conducted in the United States of America which has demonstrated that iron folate supplementation dramatically reduces the incidence of LBW infants, which is 4% in the treatment group and 17% in the placebo group [12].

Furthermore, this finding agrees with a study done in the Silte zone and Sidama regional state in South Ethiopia [2, 13]. This could imply that women who experience blood loss during menstruation or from many pregnancies could develop iron deficiency anemia; iron deficiency anemia can also result from a diet low in iron. Anemia can occur in pregnant women because the developing fetus needs the mother's iron to form red blood cells and other tissues. It was also discovered that taking iron supplements while pregnant protected term LBW babies [1].

This study showed that the mothers who have been gestational age less than 37 weeks or preterm delivered were 7.49 times more likely to face LBW neonate when compared to those participants who were delivered at a gestational age greater or equal 37 weeks or term. Additionally, according to the WHO, preterm accounts for almost one-third of LBW babies [14]. This result of the study was also supported by a study conducted in six low- and middle-income countries (Democratic Republic of Congo, Kenya, Zambia, Guatemala, India, and Pakistan) using data analyses of the Global Network's (GN) population-based registry of pregnant women and their babies in rural communities [14]. The reason for this could be recognized as the gestational age of the fetus falls below the acceptable range of time, and the body weight of the fetus falls dramatically due to prematurity. This study shows that participants who had not been informed about pregnancy-related danger signs during ANC follow-up had a 4.62 times chance of facing LBW. The reason might potentially hinder mothers' healthcare-seeking behaviors, health service utilization, and decision-making in all aspects of life. This obviously might lead to having LBW babies. Surprisingly, this finding was not much revealed by other studies.

Moreover, this study revealed that the mothers who had not experienced any danger signs during recent pregnancy were less likely to give LBW. This finding was in line with a study done in Addis Ababa, Central Ethiopia [15]. A possible explanation for the significant association was that most women with such danger signs of pregnancy (vaginal bleeding, fever, and severe abdominal pain) can result in decreasing nutrition and oxygen perfusion to the fetus which leads to LBW or death.

This study demonstrated that newborn mothers who had not faced premature rupture membranes during recent pregnancy were 73% less likely to face LBW as compared to their counterparts. This finding is supported by a study done in Tshwane District, South Africa, and in Amhara region, Ethiopia [16, 17]. The justification behind it could be the loss of amniotic fluid restricts fetal growth and results in the birth of a prematurely born which leads to LBW infant.

Finally, this study showed that mothers who experienced preeclampsia were identified as another determinant of LBW. Newborn mothers who were faced with preeclampsia during their pregnancy were more prone by 34% when compared with their counterparts. This finding is consistent with studies done in Iran and China [18, 19]. This may be associated with oxygen and nutrients supplied through the placenta to the fetus becoming compromised as a result of vasoconstriction of blood vessel walls during a hypertensive state, and uteroplacental insufficiency causes intrauterine growth retardation, resulting in LBW. Strengths of the current study include weighing the newborn within an hour of birth and taking several maternal nutrition-related factors into account. Nonetheless, there are certain limitations to the study, such as the exclusion of private health institutions. Furthermore, even though we made an effort to minimize biases by reminding participants to start with the closest meal and their last menstrual date by the calendar, there may still be recall bias with relation to dietary diversity, gestational age, number of ANC visits, and number.

## 5. Conclusion

Preterm birth, history of experiencing preeclampsia, PROM, missing of iron-folate supplementation during pregnancy, lack of information for danger signs of pregnancy, and history of any pregnancy complication were predictors of LBW. Preventing physical trauma or its possible causes throughout pregnancy, being aware of the signs and symptoms of pregnancy difficulties, and promptly identifying the causes and preventing early delivery are all advised in order to prevent LBW. LBW and its associated short- and long-term effects could be decreased by identifying high-risk moms, identifying risk factors early on, and managing those risk factors.

## Figures and Tables

**Table 1:** Sociodemographic characteristics of the mothers at Bishoftu health facilities (2022).

**Variables**	**Categories**	**Cases (** **n** = 95**)**	**Control (** **n** = 190**)**
		**N** **(%)**	**N** **(%)**
Age of mothers	<20	10 (10.5)	7 (3.7)
	20–24	30 (31.6)	56 (29.5)
	25–29	30 (31.6)	78 (41.1)
	30–34	17 (17.9)	34 (17.9)
	≥35	8 (8.4)	15 (7.9)

Sex of neonates	Female	54 (56.8)	92 (48.4)
	Male	41 (43.2)	98 (51.6)

Marital status	Currently married	91 (95.8)	183 (96.3)
	Divorced/separated/widowed	4 (4.2)	7 (3.7)

Educational status	No formal education	5 (5.3)	10 (5.3)
	Primary	29 (30.5)	51 (26.8)
	Secondary	36 (37.9)	69 (36.3)
	Higher Education	25 (26.3)	60 (31.6)

Mothers' occupation	Housewife	63 (66.3)	122 (64.2)
	Government employee	17 (17.9)	38 (20)
	Self-employed	7 (7.4)	16 (8.4)
	Others^a^	8 (8.4)	14 (7.4)

Ethnic group	Oromo	67 (70.5)	125 (65.8)
	Amhara	21 (22.1)	44 (23.2)
	Others^b^	7 (7.4)	21 (11,1)

Mothers' religion	Orthodox	55 (57.9)	101 (53.2)
	Muslim	14 (14,7)	29 (15.3)
	Protestant	25 (26.3)	55 (28.9)
	Others^c^	1 (1.1)	5 (2.6)
	Higher education	34 (35.8)	82 (43.2)

Place of residence	Urban	73 (76.8)	152 (80.0)
	Rural	22 (23.2)	38 (20.0)

BMI category	< 18.5	3 (3.2)	2 (1.1)
	18.5–24.9	67 (70.5)	117 (61.6
	≥ 25	25 (26.3)	71 (37.4)

^a^Daily labor, merchants, and students.

^b^Gurage, Tigre, and Silte.

^c^Waqefata.

**Table 2:** Obstetric and medical-related characteristics at Bishoftu health facilities (2022).

**Variables**	**Categories**	**Cases (** **n** = 95**)**	**Control (** **n** = 190**)**
		**N** **(%)**	**N** **(%)**
Pregnancy planned	Unplanned	21 (22.1)	19 (10)
	Planned	74 (77.9)	171 (90)

Gestational age	< 37	49 (51.6)	10 (5.3)
	≥ 37	46 (48.4)	180 (94.7)

Abortion	No	75 (78.9)	148 (77.9)
	Yes	20 (21.1)	42 (22.1)

Mode of delivery	SVD	62 (65.3)	122 (64.2)
	CS	33 (34.7)	68 (35.8)

Interval of pregnancy	< 24 months	11 (24.4)	11 (9.8)
	≥ 24 months	34 (75.6)	101 (90.2)

ANC follow-up	No	4 (4.2)	3 (1.6)
	Yes	91 (95.8)	187 (98.4)

Duration of ANC visit	First trimester	52 (57.1)	151 (80.7)
	Second trimester	34 (37.4)	31 (16.6)
	Third trimester	5 (5.5)	5 (2.7)

Total number of ANC visit	No visit	4 (4.2)	3 (1.6)
	1–3 visits	62 (65.3)	75 (39.5)
	≥ 4 visit	29 (30.5)	112 (58.9)

Informed about danger sign	No	25 (26.3)	12 (6.3)
	Yes	70 (73.7)	178 (93.7)

PROM	No	69 (72.6)	178 (93.7)
	Yes	26 (28.6)	12 (6.3)

APH	No	89 (93.7)	183 (96.3)
	Yes	6 (6.3)	7 (3.7)

Chronic hypertension	Yes	1 (1.1)	1 (0.1)
	No	94 (98.9)	189 (99.5)

Preeclampsia	No	64 (67.4)	170 (89.5)
	Yes	31 (32.6)	20 (10.5)

Eclampsia	No	91 (95.8)	183 (96.3)
	Yes	4 (4.2)	7 (3.7)

Anemia	Yes	4 (4.2)	2 (1.1)
	No	91 (95.8)	188 (98.9)

DM	Yes	0 (0)	2 (1.1)
	No	95 (100)	188 (98.9)

HIV	Yes	3 (3.2)	
	No	92 (96.8)	190 (100)

**Table 3:** Nutritional and substance use among mothers of LBW cases and controls.

	**Category**	**Cases**	**Control**
Smoking habit	No	93 (97.9)	187 (98.4)
	Yes	2 (2.1)	3 (1.6)

Chewing chat	No	79 (83.2)	163 (85.8)
	Yes	16 (16.8)	27 (14.2)

Alcohol consumption	No	49 (51.6)	102 (53.7)
	Yes	46 (48.4)	88 (46.3)

MUAC	< 23 cm	29 (30.5)	27 (14.2)
	≥ 23 cm	66 (69.5)	163 (85.8)

Iron and folate supplementation	No	16 (16.8)	12 (6.3)
	Yes	79 (83.2)	178 (93.7)

Dose of iron and folate	< 90 days	62 (65.3)	38 (20.0)
	≥ 90 days	17 (17.9)	140 (73.7)

Hemoglobin	< 11 g/dL	5 (5.3)	8 (4.2)
	≥ 11 g/dL	90 (94.7)	182 (95.8)

**Table 4:** Determinants of LBW in logistic regression analysis for newborns delivered in Bishoftu health facilities (2022).

**Variables**	**Categories**	**Cases (** **n** = 95**)**	**Control (** **n** = 190**)**	**COR (95% CI)**	**AOR (95% CI)**	**P.V**
		**N** **(%)**	**N** **(%)**			
Age of mothers	< 20	10 (10.5)	7 (3.7)	2.68 (0.74, 9.75)	0.58 (0.06, 5.65)	0.637
	20–24	30 (31.6)	56 (29.5)	1.00 (0.38, 2.64)	0.27 (0.05, 1.40)	0.120
	25–29	30 (31.6)	78 (41.1)	0.72 (0.28, 1.88)	0.45 (0.10, 2.12)	0.312
	30–34	17 (17.9)	34 (17.9)	0.94 (0.33, 2.64)	0.29 (0.05, 1.56)	0.148
	≥ 35	8 (8.4)	15 (7.9)	1	1	

Pregnancy	Unplanned	21 (22.1)	19 (10)	2.55 (1.30, 5.03)	1.41 (0.35, 5.66)	0.627
	Planned	74 (77.9)	171 (90)	1	1	

Gestational age	< 37	49 (51.6)	27 (14.2)	6.43 (3.63, 11.40)	7.49 (3.23, 17.36)	0.000
	≥ 37–41 weeks	46 (48.4)	163 (85.8)	1	1	

Informed about danger sign	No	25 (26.3)	12 (6.3)	5.30 (2.52, 11.12)	4.65 (1.37, 15.75)	0.014
	Yes	70 (73.7)	178 (93.7)	1	1	

Experienced danger sign	No	52 (54.7)	170 (89.5)	0.14 (0.08, 0.26)	0.21 (0.07, 0.57)	0.002
	Yes	43 (45.3)	20 (10.5)	1	1	

PROM	No	69 (72.6)	178 (93.7)	0.18 (0.09, 0.37)	0.27 (0.08, 0.91)	0.035
	Yes	26 (27.4)	12 (6.3)	1	1	

Preeclampsia	No	64 (67.4)	170 (89.5)	0.20 (0.11, 0.38)	0.34 (0.13, 0.88)	0.026
	Yes	31 (32.6)	20 (10.5)	1	1	

MUAC	< 23 CM	29 (30.5)	27 (14.2)	2.16 (1.17, 3.98)	2.74 (0.99, 7.61)	0.053
	≥ 23 days	66 (69.5)	163 (85.8)	1	1	

Dose of iron folate	< 90 days	62 (72.1)	38 (21.3)	9.52 (5.26, 17.21)	12.16 (5.40, 27.42)	0.000
	≥ 90 days	24 (27.9)	140 (78.7)	1	1	

## Data Availability

The datasets analyzed during the current study are available from the corresponding author on reasonable request.
